# Application of Fraser’s “Practical” Ethic in Veterinary Practice, and Its Compatibility with a “One Welfare” Framework

**DOI:** 10.3390/ani8070109

**Published:** 2018-07-03

**Authors:** Anne Fawcett, Siobhan Mullan, Paul McGreevy

**Affiliations:** 1 Sydney School of Veterinary Science, University of Sydney, Camperdown, NSW 2006, Australia; paul.mcgreevy@sydney.edu.au; 2 School of Veterinary Sciences, University of Bristol, Langford House, Langford BS40 5DU, UK; Siobhan.mullan@bristol.ac.uk

**Keywords:** practical ethic, One Welfare, animal ethics, ethical frameworks, veterinary, veterinary education, ethically challenging situations

## Abstract

**Simple Summary:**

Ethically challenging situations are common in veterinary practice. Veterinary ethics is considered important by veterinary students, educators, and regulators alike, and may help to reduce stress arising from ethically challenging situations in veterinarians. Ethical frameworks are designed to aid ethical decision making, but some students may find theoretical ethical terminology off-putting and may struggle to apply frameworks to real-life decisions. Fraser’s “practical” ethic is a series of principles that can be applied in ethically challenging situations. We discuss the development of this approach and apply it to examples of ethically challenging situations that veterinarians may encounter. Because Fraser’s “practical” ethic acknowledges the interdependence of animal welfare, human well-being, and the environment, we argue that it is consistent with a One Welfare framework, adopted by organisations, such as the World Organisation for Animal Health (OIE). We describe the strengths and limitations of Fraser’s “practical” ethic, and the One Welfare framework, when employed in veterinary contexts, and recommend further training and support to enable veterinarians to effectively apply these frameworks.

**Abstract:**

Ethically challenging situations are common in veterinary practice, and they may be a source of moral stress, which may in turn impact the welfare of veterinarians. Despite recognition of the importance of ethical reasoning, some veterinary students may struggle to apply theoretical ethical frameworks. Fraser developed a “practical” ethic consisting of four principles that can be applied to ethically challenging situations. We apply Fraser’s “practical” ethic to three cases that veterinarians may encounter: animal hoarding, animal neglect, and treatment of wildlife. We argue that Fraser’s “practical” ethic is consistent with a One Welfare framework, and may have increasing currency for veterinarians in the light of the World Animal Health Organisation’s Global Animal Welfare Strategy. Both Fraser’s “practical” ethic and a One Welfare framework require veterinarians to consider the impacts of animal ethics decisions on a broader scale than most other ethical frameworks have prepared them for. We discuss the strengths and limitations of Fraser’s “practical” ethic when applied in veterinary contexts and recommend additional support and training to enable veterinarians to effectively apply these frameworks in real-world settings.

## 1. Introduction

Veterinary ethics appears increasingly in veterinary curricula worldwide, and is considered to be important by students [1,2], educators [3], and registration bodies, as reflected in expected competencies [4,5,6]. The need for teaching ethical reasoning is apparent in studies where veterinarians report experiencing veterinary dilemmas commonly. A United Kingdom (UK) study of the ethical dilemmas encountered by 58 veterinary practitioners found that 57% of respondents reported facing 1–2 ethical dilemmas per week, while 34% faced 3–5 dilemmas per week [7]. In a United States (US) study of 484 veterinarians, 19 per cent reported encountering an ethical dilemma between satisfying the interests of their clients and those of their patients at least once per day [8]. Ethical dilemmas, and ethically challenging situations, may be a source of moral stress, which may impact the welfare of veterinarians [8]. Ethical reasoning did not improve with experience, suggesting that these skills should be explicitly taught within veterinary curricula [9]. 

Ethical theories are systematic attempts to explain what is right and wrong. Ethical frameworks are systems of rules, guidelines, or principles by which one or more ethical theories are applied. Teaching veterinary ethics often incorporates key ethical theories, including teleological (outcome-based) theories, such as utilitarianism, deontological (often rights-based) theories, principalism, relational ethics and virtue ethics [10,11], and ethical frameworks. This aligns with calls for students to be exposed to a range of ethical theories and frameworks, as well as examination of value systems, alternative views, conflict resolution, and decision-making processes [12].

Although these ethical theories have been applied broadly to the use of animals, none was originally developed specifically to address human-animal relations. Rather, their application to human-animal interactions and conflicts has been post-hoc. In the first author’s experience, some students struggle with the application of ethical frameworks to veterinary cases, even when they have the opportunity to workshop cases with appropriate academic support. It may be difficult for all veterinary students to achieve a nuanced understanding of theoretical ethical terminology, and some students may see different ethical theories as competing rather than complementary, leading to their becoming disenchanted with moral pluralism and what they see as relativism [13]. For these students, ethical frameworks referencing different moral theories may become a source of confusion and stress.

A discussion of the aetiology and impact of moral stress and moral distress is beyond the scope of this paper. Rather, this article discusses David Fraser’s “practical” ethic for animals [3] as an alternative framework that enables veterinarians to address, or at least to consider, concerns about animal welfare, human well-being, and environmental sustainability, while minimising the risks of unintended harm, without explicit reference to ethical theories.

In addition, we propose that Fraser’s “practical” ethic is consistent with a “One Welfare” framework. “One Welfare” was first discussed in the veterinary literature in 2013, when Colonius and Earley argued that “global ethical and policy decisions about human and animal welfare should be based on the consideration of the wellbeing of animals and humans within their ecosystem” [14]. The concept was further developed by Garcia Pinillos and colleagues [15], who positioned One Welfare as complementary to and a necessary extension of the One Health framework.

## 2. Fraser’s “Practical” Ethic for Animals 

Fraser’s term “practical” ethic alludes to Toulmin’s “practical philosophy” [16,17], which holds that abstract generalisations of theoretical philosophy often fail to provide workable guidance for complex real-world decisions. Fraser describes his “practical” ethic as a mid-level approach that is concerned with providing guidance for people, including veterinarians, making ethical decisions with practical consequences. It consists of four principles that can be applied to any case, starting from the patient or patients in our care and broadening out to the environment or ecosystem:

(1) to provide good lives for the animals in our care;
(2) to treat suffering with compassion;
(3) to be mindful of unseen harm; and,
(4) to protect the life-sustaining processes and balances of nature (a discussion of each of these principles can be found in [18]).

Fraser’s practical ethic was constructed in response to three major concerns:

(a) a recognition that human behaviour impacts animals, both intentionally and unintentionally;
(b) limitations of commonly taught ethical frameworks and concerns about their applicability to practice; and,
(c) a need to find common ground between the concerns of conservation biologists and animal welfare scientists.

We will discuss each of these areas of concern in turn.

### 2.1. Intentional and Unintentional Impacts of Human Behaviour on Animals

Fraser and MacRae (2011) identified four types of human activity that have the potential to harm animals, as well as ethical, scientific and practical challenges associated with these (outlined in Table 1).

Human actions impact animals (both positively or negatively) at different levels of biological organisation: the individual or group of individuals, the population (taxa or geographically defined), and the ecological system [18].

Fraser and MacRae argue that animal welfare scientists and animal ethics philosophers have largely been concerned with Type 1 and 2 behaviours that typically affect individual or groups of animals (often referred to as “animal use”), while ignoring Type 3 and 4 behaviours that typically (though not exclusively) impact populations and ecosystems. This reflects both a philosophical bias towards exploring the ethics of intentional acts, as well as the practical difficulty of identifying and quantifying unintentional harms, particularly those with indirect impacts (Type 4) [19]. For example, the number of birds that are killed by striking windows in the US alone is estimated to range from 100 million to one billion [20], but it is impossible to calculate with any degree of precision. In terms of the extent of impact, Fraser and MacRae argue that Type 3 and 4 behaviours often rival or exceed Type 1 and 2 activities. Thus Fraser’s “practical ethic” was designed to embrace the historically overlooked impacts of Type 3 and 4 behaviours, notably by being mindful of unseen harm (Type 3) and protecting the life-sustaining processes and balances of nature (Type 4) [18].

Unintended harm is a useful lens through which one can look at problems that might otherwise seem to produce conflict between animal rights and animal welfare approaches. Consider a situation where an animal welfare veterinarian advocates for an incremental improvement in a husbandry procedure that persons holding an animal rights view seek to ban. Being mindful of unintended harm in this situation means being mindful of potential harm done, inter alia, between (a) incremental improvement, perhaps by reducing suffering such that public concern is reduced, yet thereby unintentionally slowing reform; or, (b) through banning a procedure without establishing an appropriate alternative, leaving animals exposed to factors that the procedure was designed to protect them from [21]. For example, Australian wool has been the subject of numerous boycotts due to the practice of mulesing, which is the surgical removal of skin of the tail and breech area in sheep to prevent flystrike (see, for example [22]). While consumers and animal rights organisations, such as People for the Ethical Treatment of Animals (PETA), were frustrated by the slow progression of improvement, including the adoption of analgesia for lambs being mulesed, the ban on mulesing did not address welfare issues associated with flystrike, including pain, stress, and mortality. Thus one unintended animal welfare harm of an immediate ban on an intervention (mulesing) may be an increase in the incidence of the condition it was intended to prevent (flystrike) [23]. 

Fraser’s “practical ethic” requires that we consider which approach is likely to yield the least total harm. To this end, it employs a consequentialist approach that requires the weighing-up of harms and benefits.

Unseen, unintended harms are, by nature, challenging to predict, let alone quantify, and they may not become apparent—if at all—until after an action is taken. This is, in part, due to the complex nature of natural systems, such that “attempts to provide benefits at the level of populations or ecological systems may cause unintended harms” [18]. For example, the introduction of exotic species to benefit agricultural yields, such as the introduction of the cane toad (*Bufo marinus*) to control beetles in sugar-cane crops in Queensland Australia, has led to the decline of native species (mostly large predators), and an increase in others (mostly animals that are targeted by these predators) well beyond the sugar-cane growing regions [24]. Harms to populations or ecosystems may be indirect, taking time to manifest and making them more difficult to study.

The potential for such harm may be captured by a framework such as the ethical matrix [25], which aims to account for the interests of a broad range of stakeholders, including animals and the environment. However, Fraser explicitly cautions against “unseen harm”. The principles of the “practical ethic” require that we are open to accepting that an approach we pursue, despite the best intentions, may result in unintended harm. Thus, we are obliged to continuously review our strategy and to pursue a less harmful alternative. This is what Fraser calls a “mindfulness” toward unseen harms, which includes “noticing, articulating, and giving thoughtful consideration to such harms, reflecting on one’s own role in the harms, and having the courage to challenge existing practices” [26], and, where possible, eliminating unintended harms, while recognising that (further) unintended harms are always a possibility. Unlike the ethical matrix, which is limited to the parameters of the ethical issues raised, Fraser’s practical ethic is not similarly restricted by pre-determined parameters, as unseen harm may impact stakeholders and interests beyond those that are outlined in a matrix [27].

In this way, Fraser’s “practical” ethic is similar to the precautionary principal, which is applied to problems that are characterized by “(1) complexity in the natural and social systems that govern the causal relationships between human activities and their consequences and (2) unquantifiable scientific uncertainty in the characterization and assessment of hazards and risks” [28], which are similar to Type 3 and 4 behaviours. The precautionary principle holds that “where human activities may lead to morally unacceptable harm that is scientifically plausible but uncertain, actions shall be taken to avoid or diminish that harm” [28]. In this context, morally unacceptable harm is defined as harms to humans or the environment that are:

- threatening to human life or health, or
- Serious and effectively irreversible, or
- Inequitable to past or future generations, or
- Imposed without adequate consideration of the human rights of those affected” [28].

Like Fraser’s “practical” ethic, the precautionary principle emphasises anticipation of and the mitigation of risks. However, it does not explicitly consider animal welfare. The precautionary principle is compatible with Fraser’s “practical” ethic, in that anticipation of unseen harms may prompt a variety of precautionary actions, from not taking a particular course of action to building the resilience of an ecosystem [28].

### 2.2. Advantages of Fraser’s “Practical Ethic” in Addressing Limitations of Commonly Taught Ethical Frameworks 

Commonly taught ethical frameworks are based on fundamental principles that are then applied to real world situations in a “top-down” manner. These include frameworks that are based on utilitarianism, based on the principle that we should aim to maximise the well-being of beings (including animals) that experience pleasure and pain [29]; deontology, based on the principle that we have duties and obligations to beings deemed to have rights [30]; and, neo-Aristotelian approaches focused on respecting “natures”, allowing for beings to develop their species-specific capabilities or “telos”, and developing morally valuable, reliable character traits [11,18,31,32]. 

One prominent criticism of utilitarianism and deontology, in particular, is their inherent assumption of impartiality, as highlighted by scholars including Gilligan [33] and Midgley [34]. Such impartiality precludes us from prioritising obligations that flow from or apply to those with whom we have close relationships over obligations to others with whom we do not. As such, “rationality” takes place over “reasonableness”. Midgely argued that, “contrary to the views of some egalitarians…we can indeed owe special, over-riding duties based on kinship and other forms of social nearness. From a burning building, or even from a milder disaster, we are right to rescue first our nearest and dearest” [34]. The feminist care tradition of ethics rejects abstract, rule-based principles in favour of situational, contextual ethics, emphasising responsibility and relationships ahead of rights and rules. The problem is that an ethic of care is much harder to apply, and is not necessarily sufficient as a framework for ethical reasoning, particularly regarding the populations of animals with which we have no direct relationships, such as wildlife [35]. 

Another concern is that theoretical approaches, as “top-down” approaches, are not based on an understanding of everyday human-animal interactions. Fraser saw the need for a “bottom-up” approach that is based on induction from observation of real world human-animal interactions.

Because they are developed to be “timeless”, ethical theories are necessarily vague, attempting to espouse principles that can be applied to any issue, at any time. This may sometimes limit their applicability to current situations, which can be nuanced and contextually specific. For example, the deontological position that an ethical proposition is correct if it adheres to a universalisable moral norm becomes absurd when we apply it to real life situations. If it is always wrong to lie, then it is morally “wrong” to lie to save the life of another, regardless of the context.

Traditionally, ethical frameworks are taught with a focus on intentional actions, and intentional harm to animals, involved in intentional animal use. Yet, according to Fraser, more animals are harmed by humans unintentionally, and quite often indirectly (for example, fish killed due to pollution of waterways [19]). Of course, only those impacts that we know about can be factored into decision making. The challenge is that such harms are often unseen, and even less commonly measured. But, given the number of animals that are impacted by these unseen harms, Fraser argues that an ethical theory that fails to explicitly consider unseen harms is wholly inadequate for advancing and protecting animal welfare.

Fraser’s “practical” ethic incorporates existing ethical theories. For example, in providing good lives for animals in our care, we are maximising their welfare, which is consistent with a utilitarian approach. Providing good care for animals we interact with, is also consistent with a relational ethic or an ethic of care. Treating suffering with compassion is consistent with a virtuous approach and an ethic of care approach, as well as a deontological approach insofar as we have a duty to treat suffering. Being mindful of unseen harm reflects a utilitarian concern with consequences, in that an action cannot be good if the outcome results in overall poor well-being, as well as a virtue approach in cultivating the virtue of mindfulness. Finally, protecting the life-sustaining processes and balances of nature reflects both a utilitarian approach in maximising well-being for the greatest number of stakeholders, and an obligation to others. It incorporates elements of environmental ethics.

### 2.3. A Need to Find Common Ground between the Concerns of Conservation Biologists and Animal Welfare Scientists

Fraser identified that conservation biology, which is concerned with populations, ecological systems and taxa, and animal welfare science, which are concerned with the health and quality of life of individuals and groups of (typically) domestic animals, were often treated as distinct, and sometimes opposing, fields in the literature [26]. However, this overlooks significant common ground.

Firstly, many real-life problems, such as the use of agricultural pesticides, human infrastructure, and animal management, affect populations and ecosystems, as well as the welfare of individual animals. For example, the use of a particular pesticide may reduce a species population in a certain area, but also result in the suffering of individual members of target and non-target species [26,36].

Secondly, research methods from the fields of both conservation biology and animal welfare science may be required to meet challenges in the other. For example, a conservation program involving the trapping and translocation of animals will not be successful if the welfare of those animals is not attended to throughout and after their relocation. Similarly, animal welfare scientists may need to mark, track, and monitor free-living animals to determine the animal welfare impacts of practices of forestry, agriculture, aquaculture, and pest control [26]. Lessons learned from studies of free-ranging members of a species may be conducted primarily to inform the care of conspecifics in domestic contexts, but can also enhance appreciation of the species’ needs in the wild. 

Finally, policies and practices targeting either conservation or animal welfare are unlikely to be entirely successful if they have negative impacts on the other. For example, an intervention that results in the preservation of a species is unlikely to be acceptable if it involves subjecting surviving animals to poor welfare [26].

Additionally, Fraser notes that the conservation biology and animal welfare science face similar challenges, insofar as they are mandated or mission-oriented fields of science established to respond to societal concerns. Both fields, he argues, involve ethical, evaluative, empirical, and/or functional assumptions, and are expected to provide advice on the basis of incomplete data, where interpretation is often contentious [26].

## 3. Application of Fraser’s “Practical” Ethic in Veterinary Practice

Fraser’s “practical ethic” is an approach to animal ethics. It is therefore not designed to specifically address scenarios relating to veterinary professional ethics (for example, collegial relations). In this section of the current article, we present three scenarios that may impact welfare or wellbeing of stakeholders at various levels, and applied Fraser’s “practical” ethic to each.

### 3.1. Scenario 1: Animal Hoarding

Your client, Mr. Smith, accidentally leaves his wallet at the practice just before closing time. He is a pensioner and full-time carer for his wheelchair-bound wife. His mobility is reduced due to diabetic neuropathy. Given that the Smiths live within a short drive of the practice, you decide to drop the wallet off on your way home. The Smiths have been regular clients for 18 years, so you feel that it is the least you can do. You arrive at the property, but no one answers the front door. You notice the garage door is open and suspect Mr. Smith is in there. As you enter the garage you are hit by a nauseating smell of excrement. The garage is stacked with metal cages full of dogs—you estimate at least 20—matted, soiled, and in poor body condition, all barking. The noise is almost deafening. Mr. Smith rushes in, quickly ushers you out, and pulls the door shut. At the door of his house, Mr. Smith thanks you for the wallet, but it is clear he wishes you to leave the property. Over his shoulder you catch a glimpse of Mrs. Smith, sitting in a room filled floor to ceiling with newspapers. You cannot imagine she could navigate the house in her wheelchair. You believe that Mr. Smith (and potentially Mrs. Smith) are hoarding animals. What should you do? (Scenario adapted from Joffe and colleagues. [37]). The principles of Fraser’s “practical” ethic in relation to this case are outlined in in Table 2.

**Possible way forward:**

Once aware of the suffering of these animals, the veterinarian has a duty to act. Given the complexity of the problem, animal hoarding requires a multi-agency response [43]. This may involve contacting local organisations, such as the local council, social workers, and utility companies. The veterinarian may discuss the animal welfare problems noted with Mr. Smith and ask Mr. Smith why he is struggling to meet the welfare needs of the animals. At this point, Mr. Smith may be open to assistance. However, he may equally be evasive or defensive. The veterinarian can explain the welfare needs of the dogs and suggest how these may be met—including the surrender of the animals to an animal welfare organisation. The veterinarian can also discuss risks to Mr. and Mrs. Smith, and their neighbours, posed by zoonotic disease, pollution, fire hazards, and so on. Ultimately, if Mr. Smith is not willing to alter this situation, reporting the case to the RSPCA or equivalent may be required to prevent further negative impacts on the welfare of the animals, the Smiths and their neighbours. Where possible, the veterinarian should stay in touch with the Smiths, but there may be a role conflict if the veterinarian becomes a witness in any prosecution against them.

Animals that are removed from hoarding situations should be screened for infectious diseases and quarantined from other animals to minimise transmission of infectious diseases. Dogs that are rescued from hoarding situations should be assessed carefully before being placed in homes, in order to minimise the risk of harming future owners either through aggressive or fearful behaviour, or simply by a poor human-animal bond.

### 3.2. Scenario 2: Farmer Neglecting Stock

You are a government-employed veterinarian called to investigate a report of neglected cattle on a property. When you attend the property, you note that the paddocks are overrun with weeds. The fences are in disrepair. Cattle are in poor condition, and you find two dead animals in a stream at the border of the property. The owner takes some time to rouse and appears to be disheveled. He agrees with you that the stock are in poor condition, but does not seem equipped to address the issues. You ask about the cattle in the water course and he says that he tried to remove the carcasses but with no assistance this was impossible. He explains that he lost his wife to cancer twelve months ago. You suspect he has a substance abuse problem. The principles of Fraser’s “practical” ethic in relation to this case are outlined in in Table 3.

**Possible way forward:**

Mental health problems can compromise the ability of farmers to function rationally, and therefore maintain proper welfare standards on the farm [44]. The veterinarian may experience a conflict between the desire to assist and educate the farmer, and to improve animal welfare this way, or the requirement to escalate the complaint.

It is important that both animal welfare and farmer mental health issues are addressed so that these issues do not recur. In such a case, it is unlikely that the farmer currently has the capacity to care for these animals. Ideally, they should be relocated to an appropriate property, cared for by a third party (for example, with supplementary feeding), or slaughtered humanely if this cannot occur. The carcasses must be removed from the water course immediately.

It is likely that the farmer would be charged for animal welfare related offences, which is a stressor that may worsen their mental health. The veterinarian can ask the farmer if they can contact a third party to provide support, such as a local drug and alcohol counselling service. They can also ask if they can contact local groups that can assist e.g., in providing on-farm labor while the farmer seeks treatment, or someone who is well-known to the farmer, to provide support.

A time frame should be given for rectification of the issues, to enable the farmer to address concerns, but also to ensure that animal suffering is minimised. Provision should be made for the ongoing monitoring of the property, and regular discussions with the farmer on a regular basis to ensure that animal welfare standards are not slipping. 

### 3.3. Scenario 3: Treatment and Release of Wildlife 

You work in an urban based practice in Darwin, Australia. A member of the public brings an injured common Brushtail possum (*Trichosurus vulpecula*) that was found clinging to the base of a tree after a storm. Aside from some superficial cuts the animal (a juvenile male) appears to be in good physical condition but will need to stay in hospital for 48 h until a carer can collect him. Common brushtails are overabundant in the city, but are rare in native habitats [45,46]. The principles of Fraser’s “practical” ethic in relation to this case are outlined in in Table 4.

**Possible way forward:**

Animals that are going to be released into the wild should be screened for key infectious diseases to ensure that they are not a threat to the wild population. If the possum is healthy, then it should be released to an experienced wildlife carer as early as possible to minimise stress from hospital. Ideally, it would be helpful if the carer is linked to a conservation program, as it may be possible to subsequently release the animal into an area where possums are not over-abundant. The animal should be monitored following re-release, as its fate may determine the site of release of other possums. Although the veterinarian may have less involvement once the animal is in care, he or she can talk to wildlife veterinarians about proper assessment and screening of animals in their care. If the possum is euthanased, then the veterinarian should provide post-mortem samples to the local wildlife disease association for examination. Veterinarians can make themselves aware of carers and conservation programs and alert these groups to any trends regarding admitted wildlife. 

### 3.4. Discussion

While not initially designed to be applied in a table format, the application of the four principles lends itself to tabulation. While the first and fourth principles apply to dependent animals and the environment (including animals in the wild), we have interpreted the second and third principles as requiring that we consider suffering of and unseen harms to human and animal stakeholders. For this reason, listing all such stakeholders is a useful first step. For example, stakeholders in the first scenario include Mr. and Mrs. Smith, the dogs, the veterinarian, the neighbours, the local environment, any of whom or which may be negatively or positively affected by decisions made. Some, such as the dogs and the Smiths, may be impacted directly, and some indirectly. Similarly, unseen harm may apply to the Smiths, the environment, or other stakeholders.

As with the ethical matrix, the more comprehensive the knowledge base of the decision-maker, the more comprehensively the impacts (intended and unintended, seen and unseen) can be considered, and the better the quality of the decision ultimately made. In applying these principles, other ethical approaches are incorporated. For example, where laboratory animals are concerned, laboratory animal legislation and codes of practice typically incorporate the 3Rs of replacement, reduction, and refinement [48]. This is a utilitarian framework that requires us to weigh-up costs (in terms of welfare) against benefits (typically the benefit of advancing scientific knowledge, usually to humans). Similar approaches have been advocated for the ethical use of horses in equitation [49] and racing [50].

Unseen and unintended harms can be difficult to predict or even detect. Addressing this requires an openness to the possibility of such harms and necessitates pre-emptive steps to eliminate or minimise the risk of known potential harms. Consequentialist approaches including utilitarianism incorporate known harms in outcome assessment, but not necessarily unseen harms. Fraser’s “practical” ethic explicitly obliges us to plan for ongoing monitoring and review of outcomes. 

As with any ethical framework, the nature and the scope of factors feeding into each of Fraser’s principles depends on the knowledge, values, and biases of the people applying them, variables that will impact the overall quality of the decision made. Application of the principles alone does not generate the “right” decision. Different people applying the same principles may come to different decisions. But, Fraser’s “practical” ethic does ensure that a decision considers potential impacts on animal welfare, human wellbeing and environmental sustainability.

Mid-level principles are intended to provide guidance for action in the context of practice. However, they do not address theoretical questions, such as whether people should own or use animals in the first place. Such questions may be best addressed using ethical theories, such as utilitarianism or deontology. 

## 4. How Does Fraser’s “Practical” Ethic Relate to a “One Welfare” Framework?

According to Fraser, the term “One Welfare” is used to “emphasise the many links between animal welfare and human welfare, and to acknowledge that both depend on a well-functioning ecological environment” [51]. It is “a unifying concept for different areas of research and action, and a call to consider human welfare, animal welfare and environment together” [51].

In 2017, the World Organisation for Animal Health (OIE) developed its Global Animal Welfare Strategy, aiming for “a world where the welfare of animals is respected, promoted and advanced, in ways that complement the pursuit of animal health, human well-being, socio-economic development and environmental sustainability” [52]. The Strategy acknowledges the close and often inextricable links between animal welfare, animal health, the health and well-being of people, and the sustainability of socio-economic and ecological systems [52]. 

By applying each principle to our interactions with animals, Fraser’s approach requires us to consider the welfare of individual animals that are directly involved, the well-being of human stakeholders (both direct, such as animal owners, and indirect, such as members of the community) and the environment. Thus, it elegantly gels with the One Welfare framework, which also requires us to consider not only Type 1 and 2 impacts on animals, but also Type 3 and 4.

The “practical” ethic also tends to harmonize animal ethics and conservation within a single framework rather than having them as separate or competing interests. Rather than trying to classify a problem as a conservation problem or an animal welfare problem, Fraser’s view is that they are different levels from which to look at the same problem. This is why Fraser’s “practical” ethic requires us to act at the level of the individual animal (to provide good lives for animals in our care and to treat suffering with compassion, to be mindful of unseen harm (to individuals)), but also more broadly to be mindful of unseen harm (to others) and to protect the life-sustaining processes and the balances of nature [18]. In the veterinary clinical context, the impact of decisions on animal welfare and wellbeing of clients is usually explicitly considered. But, asking the questions “how could this decision lead to unseen harms?” and “how might this decision impact the environment?” may ensure that decisions are made within a One Welfare framework.

Consider an animal welfare charity that has been awarded a grant to construct a state-of-the-art animal shelter and rehoming facility. Fraser’s principles require us to consider the design of the facility not only from the level of the animals that will be housed within it, staff and volunteers working there, and potential animal adopters, but also the wider community and the environment in which it is situated. Similarly, animal welfare policy that negatively impacts food costs and accessibility, animal habitats, and pollution (for example, through increasing energy use), would not be acceptable.

The requirement to consider the needs of the widest range of stakeholders highlights the need for thoughtful planning and humility as reflective practitioners must be prepared to acknowledge oversights and to place their decisions under an ongoing watching brief. 

## 5. Challenges for Veterinarians in Practice 

Veterinarians in practice settings are accustomed to interactions with animals and clients, and therefore may be expected to be adept at implementing the first two principles of Fraser’s “practical” ethic. 

To the extent that they monitor the health and welfare outcomes of patients, they may also be mindful of unseen and unintended harms (the third principle), however the application of this principle should extend beyond the consideration of harms to patients and clients, to broader populations and the environment. This may be challenging for the time-poor practitioner.

Both Fraser’s “practical” ethic and a One Welfare approach require veterinarians to consider the impacts of animal ethics decisions on a broader scale than most other ethical frameworks have prepared them for [53]. Their triple bottom lines should not be taken to mean that each of the elements is equally weighted. The weighting of each principle may vary between cases. For example, the fourth principle, protecting the life-sustaining processes and balances of nature, may be of minor consequence in ethical decision making around routine husbandry procedures in companion animals. However, it may remain a helpful exercise to consider the environment in these cases. 

Individual veterinarians may a struggle to implement the third and fourth principles, as it may be difficult for them to detect unseen harms of actions and interventions (for example, the long-term impact of keeping an unusual or exotic species on public attitudes to this species), or indeed to address environmental impacts (such as the impact of medical waste generated by a particular intervention on the environment, or the impact of antimicrobial residues in waterways). As noted by Ropohl, “the lack of universal expertise is a fundamental constraint of individual responsibility” [54]. To this end, veterinarians need to work with their professional associations, governments, NGOs, and even private enterprise, such as pharmaceutical companies and vaccine manufacturers, to explore and monitor broader, unintended impacts of veterinary practice, so that that strategies can be developed to minimise these. This will require additional training in environmental sustainability and ecology, and the ability to collaborate with environmental scientists and ecologists.

Veterinary ethics tends to consider the veterinarian as an autonomous actor, who is able to consider the consequences of their actions, and to act in a way that brings about what they deem to be right, and to prevent what they deem to be wrong. However, veterinarians typically work in teams and many are dependent on employers, so their autonomy is mitigated. Ethical codes that ignore the limited acting competence of individual veterinarians may not be helpful. Rather, institutional support and support at the level of the profession are required to complement and strengthen the efforts of individual veterinarians [54]. 

There are currently no studies assessing the impact of application of particular ethical frameworks on moral stress, or moral distress, in veterinarians. It is possible, for example, that ethical frameworks like Fraser’s “practical” ethic expand veterinarians’ sense of personal responsibility, and potentially increase moral stress and moral distress. Alternatively, if such a framework enables veterinarians to make decisions that are aligned with their values, it may decrease moral stress and moral distress. There is scope for further study in this area. 

Fraser’s principles provide a broad checklist that may have the added benefit of appealing to veterinary students who are confused or disenchanted with theoretical ethical terminology and current frameworks. Further studies are required to explore the use of Fraser’s “practical” ethic in veterinary ethics teaching.

## 6. Conclusions

Fraser’s “practical” ethic can be applied to animal ethics decision-making in a veterinary context. It may be useful in veterinary education, alongside other ethical frameworks, and as an aid to ethical decision making in veterinary settings. It is compatible with a One Welfare framework, which considers animal welfare, human well-being, and environmental sustainability. In addition, Fraser’s “practical” ethic explicitly calls for consideration of unseen and unintended harms. This is important for veterinarians as ethical decisions regarding animals may have short- and long-term consequences. It requires veterinarians to be mindful that current practices may be identified as harmful as further evidence comes to light. Further studies are required to explore the use of Fraser’s “practical” ethic in veterinary ethics teaching.

## Figures and Tables

**Table 1:** Types of human activity with the potential to harm animals, and their associated ethical, scientific, and practical challenges.

Type of Activity	Examples	Ethical Challenges	Scientific and Practical Challenges
1. Keeping animals	Keeping and using companion, farm, laboratory or captive wild animals.	Determining what form and level of care is ethically appropriate. Determining how to decide to rehabilitate or euthanase wild animals. Deciding whether a high level of care/welfare justifies forms of animal use (e.g., labor and food production).	Finding and adopting better animal care practices that also benefit keepers. Identify and address economic and other barriers to appropriate animal care.
2. Causing deliberate harm to animals	Slaughter, pest control, hunting, animal-based research (e.g., toxicology testing)	Deciding when deliberate harm is justified. Determining how to weigh costs to animals against benefits to people.	Developing strategies to minimise harm while achieving desired outcomes.
3. Causing unintended harm to animals	Bird deaths caused by windows, animals killed by motor vehicles, wind farms, crop production, night-time lighting.	Incorporating unintended harms into ethical decision making.	Identifying the scope and severity of unintended harms. Meeting human objectives while minimising such harms.
4. Harming animals indirectly by disturbing ecological systems and processes of nature	Pollution, anthropogenic climate change, introduction of foreign species, soil erosion, habitat destruction.	Including harms to animal welfare as well as threats to human interests and conservation.	Identifying interventions that are positive for animal welfare, conservation and human well-being.

Adapted from [19].

**Table 2:** Fraser’s “practical” ethic principles applied to animal hoarding scenario.

Principle	Specific Considerations
Provide good lives for animals in our care	Whilst you have not been called to the property to attend to any animals, you have become aware that there are at least 20 dogs kept in poor conditions and suffering as a result. In most jurisdictions, veterinarians are required to provide immediate care (including first aid, timely referral or euthanasia) of animals that are suffering (for example, see the NSW Veterinary Practioner’s Code of Professional Conduct [38]). The animals require immediate veterinary attention which the Smiths are clearly not providing. The veterinarian must consider whether they offer to treat the animals and help the Smiths meet the welfare needs of the animals (and indeed whether the Smiths have the resources to do this) or whether to report the Smiths to local authorities for animal hoarding related offences. This may result in seizure and subsequent treatment or euthanasia of the dogs. In addition, it may involve prosecution of the Smiths for animal hoarding related offences. The current situation does not meet the welfare needs of the animals and shows a deficit in care which must be addressed.
Treat suffering with compassion	The suffering of the animals should be considered. In addition, it is likely that Mrs. Smith’s needs are not being met in the current situation where objects are also hoarded. Mr. and Mrs. Smith too may be suffering from psychological morbidity [39]. As such any communication with them should be sensitive, with involvement of healthcare professionals such as social workers and carers as soon as possible. While the animals are living in terrible conditions, the owners are too. Attending veterinarians should refrain from being judgmental, as they are most likely to be able to help both the animals and the Smiths if they can maintain a positive relationship.
Be mindful of unseen harm	Aside from the obvious concerns, potential unseen harms include but are not limited to: Spread of infectious diseases to humans and other animals [40,41], causing harm to humans and animals involved in the hoarding situation, as well as humans and animals in contact with these; Behavioural abnormalities [42], which may harm affected animals as well as any other humans or animals they are subsequently homed with. Recidivism rates for animal hoarding are high, so veterinarians should try to openly communicate with persons involved in animal hoarding. Recidivism in these cases will harm additional animals. Reduction in the Smiths’ trust of the veterinarian and the profession, especially in situations where the owner is reported to the authorities by a veterinarian. These owners may be less likely to seek veterinary care for their animals in the future. Failure to address the human aspect of the problem whilst addressing the animal aspect may be detrimental to the health of the Smiths [43] and may lead to recidivism and harm of more animals.
Protect the life-sustaining processes and balances of nature	Animal hoarding impacts the environment, for example by concentrating infectious agents in the environment, creation of pollution (particularly noxious odors such as ammonia), attracting vermin and insects and preventing access to utilities [37]. Local councils may become involved with clean-up of hoarding properties which otherwise impact humans, domestic animals and wildlife species living nearby.

**Table 3:** Fraser’s “practical” ethic principles applied to farmer neglecting stock scenario.

Principle	Specific Considerations
Provide good lives for animals in our care	The veterinarian can give specific and practical animal health care advice, nutritional advice and pasture assessment (or seek advice from another suitably qualified person to do so). The veterinarian can express concerns about the ongoing care for these animals and provide practical assistance or connect the owner with agencies who can provide practical assistance, to facilitate appropriate animal husbandry.
Treat suffering with compassion	The veterinarian can listen to the farmer and try to find out when things went wrong, what resources the client has access to and what are the key barriers to appropriate animal care. Euthanasia may be required for animals in poor condition. Alternatively, arrangements may be made to sell animals if they are fit to transport, to ensure they are relocated to a property where their welfare needs can be met. A time-frame can be given to enable the farmer to rectify the issues. The farmer must be dealt with sensitively, as the welfare of animals in his care depends on his well-being. The veterinarian can provide information regarding health care support resources and services and ask the farmer if he or she can contact a mental health service on his behalf.
Be mindful of unseen harm	In relocating animals, it is important to ensure that the property they are relocated to is suitable and not overcrowded, and that these animals do not introduce disease to other animals (or vice versa). If the complaint is escalated, the client’s mental health may worsen. Farmers charged with and convicted for animal neglect or cruelty face stigmatisation, which may be exacerbated by media reporting [44]. This may add to pressures contributing to mental health problems associated with poor animal care.
Protect the life-sustaining processes and balances of nature	It is important to address the health of the animals, as a poorly managed herd can have environmental impacts on paddock health, parasite load, erosion and land degradation and interaction of domestic with wildlife. Dead animals contaminate water courses which flow onto other properties, and these should be removed as soon as possible. The weeds should be removed, and paddocks cycled so that they can be used for other animals. It is also important to ensure that the cattle are moved to an appropriate site with appropriate stocking density.

**Table 4:** Fraser’s “practical” ethic principles applied to wildlife treatment scenario.

Principle	Specific Considerations
Provide good lives for animals in our care	Hospitalised possums require appropriate feeding (including native plant species), enrichment, and monitoring to ensure their health does not deteriorate, and treatment for any diseases/injuries.
Treat suffering with compassion	Capture is stressful to possums and has been shown to cause immunosuppression, leading to disease [47]. Common brushtail possums are nocturnal, arboreal wild animals that are predated by dogs and cats. This animal is likely to have been highly stressed when found and may be very stressed in the veterinary hospital setting where the sights, scents and sounds of predators are concentrated. Keeping possums singly housed, minimising handling (for example weighing animals in their denning sack) and conditioning them to approach the carer by offering fruit may reduce stress, though it took animals 29 days to approach the caregiver in one study [47].
Be mindful of unseen harm	Care should be taken when releasing wildlife. Possums are territorial and may fight, leading to injuries/immunosuppression and increased risk of disease. Furthermore, releasing possums in an area where the species is already overabundant leads to resource competition and environmental damage. The fate of released possums should be monitored where possible to ensure that they are able to recover.
Protect the life-sustaining processes and balances of nature	Possums are opportunistic omnivores, feeding on the eggs and chicks of native birds and native insects, as well as causing damage to (and local extinction of) native plants by selective browsing [47]. In New Zealand, they are known to spread *Mycobacterium bovis* (responsible for bovine tuberculosis), which impacts the dairy and beef industries and leads to wastage of animals. Possums should not be released into sites where they are overabundant.
